# IS NARCISSISTIC PERSONALITY ASSOCIATED WITH SUICIDE IDEATION IN MIDDLE-AGED AND OLDER MEN?

**DOI:** 10.1093/geroni/igac059.1471

**Published:** 2022-12-20

**Authors:** Marnin Heisel, Gordon Flett, Paul Links

**Affiliations:** The University of Western Ontario, London, Ontario, Canada; York University, Toronto, Ontario, Canada; McMaster University, Hamilton, Ontario, Canada

## Abstract

Older adults have high rates of suicide, necessitating theory and research on factors that contribute to suicide risk and may be targets for intervention. Clark (1993) theorized that age-related losses and transitions can trigger narcissistic injury in older men, and lead to mood decline, substance misuse, loss of insight, and suicide ideation and behavior. We tested elements of Clark’s model in a secondary analysis of a geriatric depression clinic database (n=574), and reported a positive association between clinician-diagnosed Narcissistic Personality (NP) and suicide ideation and behavior, controlling for depression severity (Heisel et al., 2007). We sought to replicate and extend these findings in 82 community-residing men, 55 years and older (M=63.3, SD=4.6), who participated in a trial of Meaning-Centered Men’s Group (MCMG; Heisel et al., 2020) for those concerned about or struggling with the transition to retirement. Participants completed the Pathological Narcissism Inventory (Pincus et al., 2009), Geriatric Suicide Ideation Scale (Heisel & Flett, 2006), and Geriatric Depression Scale (Yesavage et al., 1983). Linear regression analyses indicated a significant association between PNI-Contingent Self-Esteem and GSIS totals (t=2.41, p=.019) and GSIS Loss of Personal Worth (t=2.10, p=.040) and Perceived Meaning in Life Subscales (t=2.80, p=.007), controlling for depressive symptom severity (Geriatric Depression Scale; Yesavage et al., 1983), suggesting an association between suicide ideation and self-esteem issues. These and other findings will be discussed in a broader thematic context regarding masculinity, loss, life transitions, and suicide prevention.

